# Supplementary material for: The interactome of intact mitochondria by cross-linking mass spectrometry provides evidence for coexisting respiratory supercomplexes
Source: Mol Cell Proteomics. 2017 Dec 8;17(2):216–32. doi: 10.1074/mcp.RA117.000470 (PMC5795388; doi:10.1074/mcp.RA117.000470)
Supplement: Supplemental Data [file supp_RA117.000470_133922_0_supp_23978_fzffwf.zip › spectra_annotation/mito_DR_spectra_annotation/184-1-8-1-12-1.pdf]

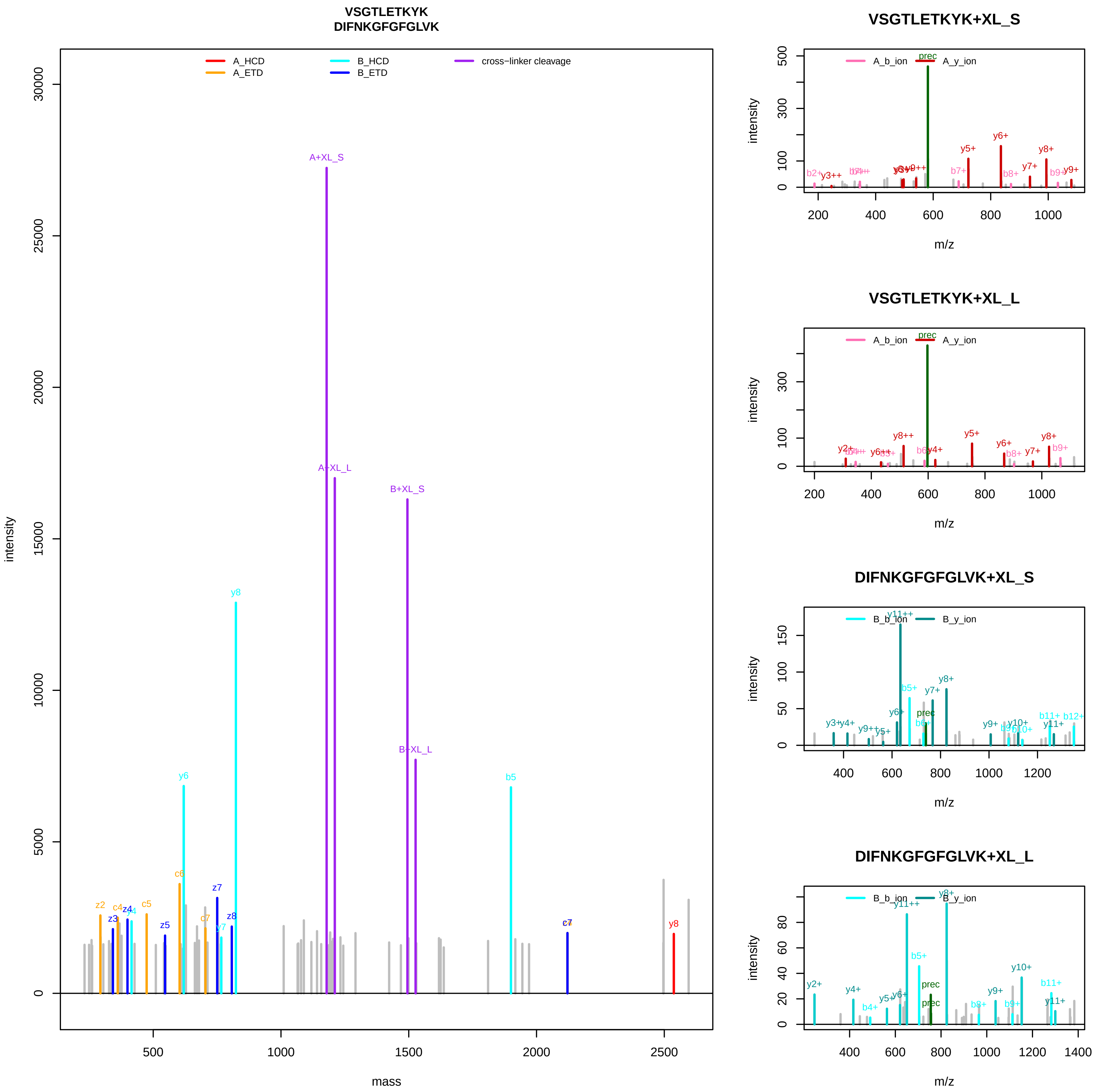

VSGTLETKYK
DIFNKGFGFGLVK
A_HCD
A_ETD
B_HCD
B_ETD
cross−linker cleavage
A+XL_S
A+XL_L
B+XL_S
B+XL_L
y8
y6
b5
c6
z7
z8
c7
y7
z5
c5
z2
z3
c4
z4
y4
y8
c7
intensity
mass
0
5000
10000
15000
20000
25000
30000
500
1000
1500
2000
2500
VSGTLETKYK+XL_S
A_b_ion
A_y_ion
prec
y6+
y5+
y8+
y7+
y9+
b7+
b8+
b9+
b2+
y3++
intensity
m/z
0
100
300
500
200
400
600
800
1000
VSGTLETKYK+XL_L
A_b_ion
A_y_ion
prec
y5+
y8++
y6+
y8+
y7+
b8+
b9+
y2+
y4+
intensity
m/z
0
100
300
200
400
600
800
1000
DIFNKGFGFGLVK+XL_S
B_b_ion
B_y_ion
y11++
b5+
y7+
y8+
y6+
prec
y3+
y4+
y9++
y5+
y9+
y10+
b10+
b11+
b12+
y11+
intensity
m/z
0
50
100
150
400
600
800
1000
1200
DIFNKGFGFGLVK+XL_L
B_b_ion
B_y_ion
y8+
y11++
b5+
y10+
b11+
y2+
y4+
b4+
y5+
y6+
prec
y9+
b8+
b9+
y11+
intensity
m/z
0
20
40
60
80
400
600
800
1000
1200
1400
